# Cost-utility analysis of palonosetron in the antiemetic regimen for cisplatin-containing highly emetogenic chemotherapy in Japan

**DOI:** 10.1186/s12913-019-4281-0

**Published:** 2019-07-01

**Authors:** Munenobu Kashiwa, Ryo Matsushita

**Affiliations:** 1 Division of Pharmacy, Graduate School of Medical Sciences, Kanazawa University, Kakuma-machi, Kanazawa, 920-1192 Japan; 2 Department of Pharmacy, First Towakai Hospital, Takatsuki, Japan; 3 Division of Pharmaceutical Sciences, Graduate School of Medical Sciences, Kanazawa University, Kanazawa, Japan

**Keywords:** Pharmacoeconomics, Cost-effective, Chemotherapy, Palonosetron, Emesis

## Abstract

**Background:**

An antiemetic triplet regimen of 5-hydrotryptamine-3 receptor antagonist, dexamethasone, and aprepitant is the standard prophylaxis with highly emetogenic chemotherapy (HEC). A randomized phase III trial comparing palonosetron (PALO) versus granisetron (GRA) in the triplet antiemetic regimen (The TRIPLE study) showed the superiority of PALO over GRA for delayed-phase vomiting in patients receiving cisplatin-based HEC. However, economic efficiency evaluations including quality of life have not been done. The present study was a cost-utility analysis of PALO within the Japanese medical insurance system.

**Methods:**

The data source was the results of the TRIPLE study. A decision tree was constructed to assess the incremental cost-effectiveness ratio (ICER) using quality-adjusted life years (QALYs) and the medical service fees and the drug price for 2018 from the perspective of the payer. A one-way sensitivity analysis and a probabilistic sensitivity analysis (PSA) were performed to assess the robustness of the model. A threshold analysis was performed to determine the cost-effective price of PALO.

**Results:**

In the base case, the estimated incremental effect of PALO addition was 0.000645 QALYs, the estimated incremental cost was 10,455 JPY (93.21 USD), and the ICER was 16,204,591 JPY QALY (144,465 USD/QALY). In the PSA, the probability of superior cost-effectiveness was 3.64%. In the threshold analysis, the acceptable price of PALO was estimated to be 7,743 JPY (69.03 USD).

**Conclusions:**

If willingness-to-pay is taken as 5,000,000 JPY/QALY (44,575 USD/QALY), the antiemetic regimen using PALO for cisplatin-containing HEC was not cost-effective at this time. The cost of drugs, with the arrival of inexpensive generic drugs, will make a major contribution to its cost-effectiveness.

## Background

Chemotherapy-induced nausea and vomiting (CINV) is a typical adverse reaction to anti-cancer agents. The frequency of CINV varies widely depending on the drugs used, and emetogenicity is classified as high (> 90%), moderate (30–90%), low (10–30%), and minimal (< 10%). The guidelines of scientific societies such as the American Society of Clinical Oncology, the National Comprehensive Cancer Network, the Multinational Association of Supportive Care in Cancer (MASCC), and the Japan Society of Clinical Oncology (JSCO) indicate that antiemetic therapy should be used according to the level of emetic risk [1–4]. CINV occurs not only on the day of chemotherapy, but also on subsequent days, and it is a major factor affecting patients’ quality of life (QoL) and continuity of chemotherapy [5].

A 5-hydroxytryptamine3 receptor antagonist (5-HT_3_RA) is now the standard therapy for preventing CINV, due to its ability to block emesis caused by the stimulation of 5-HT3 receptors on vagal afferents. The first-generation 5-HT_3_RAs, ondansetron and granisetron, dramatically changed the management of CINV in patients receiving highly or moderately emetogenic chemotherapy. Regimens that include cisplatin, which is widely used in the treatment of lung, stomach, and head and neck cancers, meet the criteria for highly emetogenic chemotherapy (HEC), and the JSCO Clinical Practice Guidelines for Antiemesis In Oncology recommend an antiemetic triplet regimen of 5-HT_3_RA, neurokinin 1 receptor antagonist, and dexamethasone (DEX) during HEC. Of these three agents, the 5-HT_3_RA palonosetron (PALO) was approved by the US Food and Drug Administration in addition to existing agents in 2003, and it was also approved for use in Japan in 2010. PALO has a plasma elimination half-life of approximately 40 h, which is longer than that of existing 5-HT_3_RAs, and it has approximately 100 times the affinity for 5-HT_3_R receptors. It is thus regarded as a second-generation 5-HT_3_RA that inhibits not only acute-phase, but also delayed-phase nausea and vomiting. The PROTECT study of a doublet regimen of 5-HT_3_RA and DEX found that, compared to granisetron (GRA), the efficacy of PALO was non-inferior for acute-phase CINV and superior for delayed-phase CINV [6]. However, since this study did not include aprepitant (APR), the results could not subsequently be directly extrapolated to everyday clinical practice. The usefulness of PALO in combination with APR and DEX was investigated in a randomized phase III trial (The TRIPLE study) [7]. This study compared PALO and GRA in an antiemetic triplet regimen of 5-HT_3_RA, APR, and DEX. In the TRIPLE study, the primary endpoint was complete response (CR; no vomiting/nausea and no rescue medication) within 120 h after cisplatin-containing HEC. Eligible patients were randomly assigned to double-blind antiemetic treatment with either GRA (1 mg) or PALO (0.75 mg). Although the primary endpoint was not achieved and the superiority of PALO was not shown in this clinical trial (*P* = 0.0539), PALO showed superiority to GRA in the CR rate in the delayed phase. Based on the results of this study, the JSCO Clinical Practice Guidelines for Antiemesis in Oncology recommend the use of PALO with HEC containing 50 mg/m^2^ or more cisplatin. Guidelines in other countries also recommend PALO with moderately emetogenic chemotherapy [1–3]. However, a meta-analysis by Kolesar et al. reported insufficient grounds to recommend PALO over other 5-HT_3_RAs [8], and recent guidelines have positioned PALO at the same level as other 5-HT_3_RAs [1–3]. The positioning of PALO in clinical practice is thus different from other countries in the Japanese guidelines. Japan has a national health insurance (NHI) system and depending on the age, patients have to pay 10–30% of medical expenses. Lower out-of-pocket costs are particularly beneficial for the elderly, but the increase in medical expenditures in the national budget as a result of the aging population is a serious problem. The increased economic burden is a concern for PALO, which is a more expensive antiemetic than other 5HT3RAs.

As to concerns about the economics of PALO, results of analyses in the USA and China reported that PALO has poor cost-effectiveness [9, 10]. In Japan, Shimizu et al. reported that treatment with PALO is expensive based on retrospectively investigating the costs of patients in the TRIPLE study [11]. Although the cost per vomiting control was measured, there has been no economic evaluation including QoL. In order to compare with medical technologies, analyses including QoL are required. Cost-utility analysis is a measure used for this in the economic evaluation of medical technologies. If such an evaluation is completed within a clinical trial, it is desirable from the perspective of internal validity, but there may be a problem with the generalizability of the result. To the best of our knowledge, no study has directly compared the cost-effectiveness of the prevention of CINV associated with HEC with triple therapy combining DEX and APR with either a first-generation 5-HT_3_RA or palonosetron. The present study was a cost-utility analysis based on the Japanese health care system to define the cost-effectiveness of PALO compared to GRA as the standard prophylaxis in patients who received cisplatin-containing HEC.

## Methods

### Modeling

**Fig. 1:**
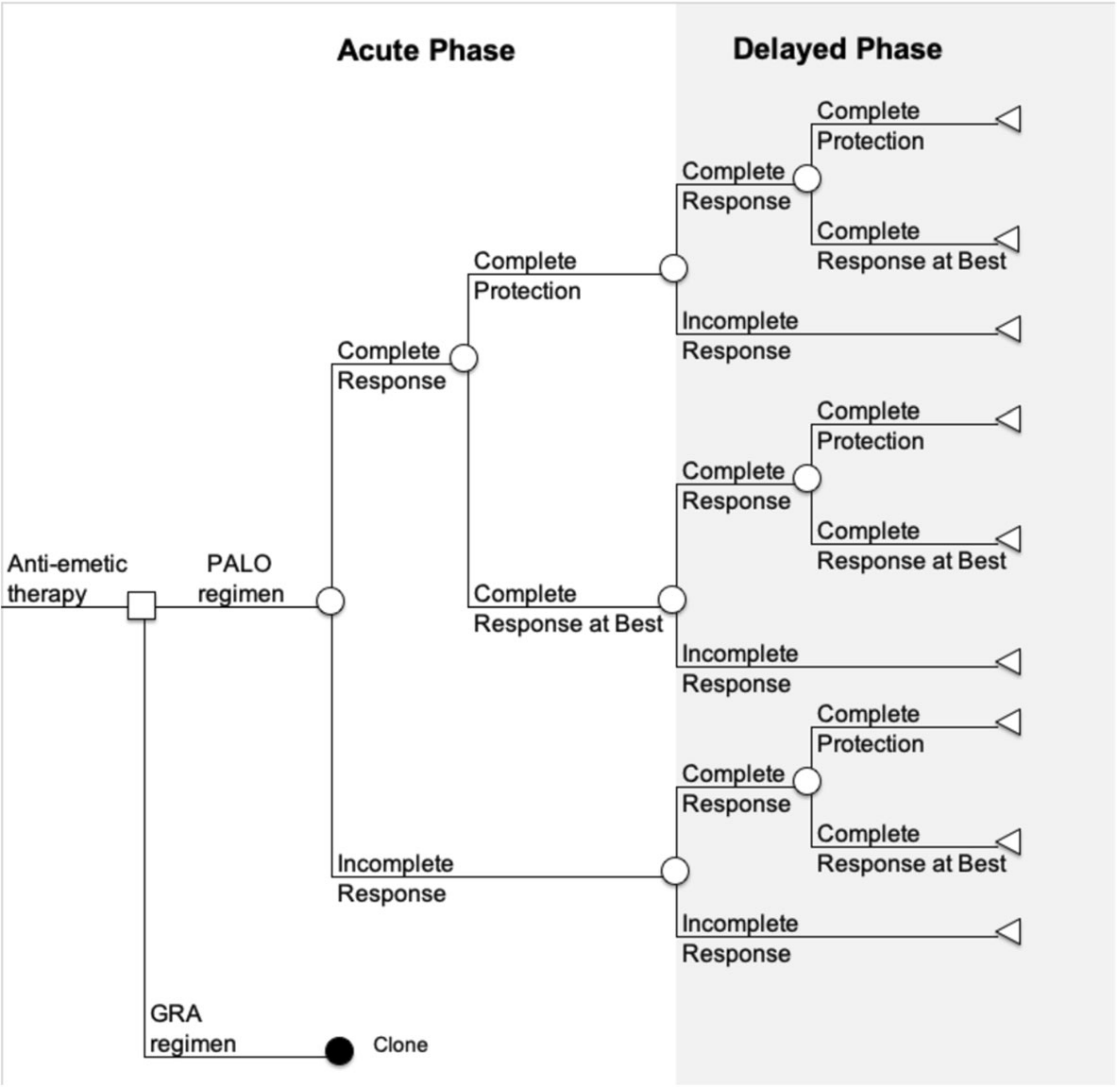
Decision tree for cost-effectiveness analysis. PALO: palonosetron; GRA: granisetron

A decision tree that showed the acute phase and the delayed phase was constructed according to prior reports [12–14] to estimate and compare the health outcomes and cost of prophylactic antiemetic therapy using PALO (Fig. 1). The clinical outcome was defined as CR with no emesis and no rescue antiemetic therapy, and incomplete response (IR) was defined as some emesis or some use of rescue antiemetic therapy. CR was subdivided into two mutually exclusive health outcomes: complete protection (CP), which was defined as no emesis, no rescue antiemetic therapy, and no significant nausea; and complete response at best (CRB), which included those who achieved CR but not CP. For the purposes of this study, it was assumed that chemotherapy was administered on an outpatient basis, and the analysis period was defined as 5 days from administration of chemotherapy.

### Clinical data

The data source was the results of a phase III study with Japanese participants (The TRIPLE study [7]. In this study, patients aged 20 years over were included if they had solid cancer and were receiving HEC containing 50 mg/m^2^ or more of cisplatin, Eastern Cooperative Oncology Group performance status of 0–2, and no organ dysfunction during the 8 days prior to enrolment. The male:female ratio of subjects was 3:1, and 70% or more had exceeded 70 mg/m^2^ of cisplatin. The tumor site was respiratory in approximately 50% of cases, followed by esophageal, gastric, head and neck, and others. A total of 842 patients were assigned to either the PALO regimen or the GRA regimen, of which the results from 414 patients in the PALO regimen and 413 in the GRA regimen were used for the analysis. The degree of nausea and vomiting and the use of rescue antiemetic therapy over the 120-h period following administration of the anti-cancer agent were examined from the patients’ symptom diaries. The primary outcome was CR over the whole period (0–120 h). For the secondary end point, CR was evaluated in the acute phase (0–24 h) and the delayed phase (> 24–120 h). The CR rates in the TRIPLE study were 59.1% (244/413 patients) for the GRA group and 67.2% (272/414 patients) for the PALO group. According to the rate of CR or complete control of acute and delayed CINV, transition probabilities were calculated by multiplying the probability of each state (CP, CRB, and IR) using the data from the TRIPLE study results (Table 1).

**Table 1:** Health state probabilities used in the model, based on the TRIPLE study

Health state outcome by phase		PALO regimen *N* = 414 (%)	GRA regimen *N* = 413 (%)
Acute Phase (day 1)	Delayed Phase (days 2–5)		
Complete protection	Complete protection	58.7	50.4
	Complete response at best	1.8	2.9
	Incomplete response	29.6	36.9
Complete response at best	Complete protection	1.1	1.0
	Complete response at best	0.0	0.1
	Incomplete response	0.6	0.7
Incomplete response	Complete protection	5.3	4.6
	Complete response at best	0.2	0.3
	Incomplete response	2.7	3.4

### Treatment strategy

Prophylactic antiemetic triplet regimens of a 5-HT_3_RA, DEX, and APR (125 mg on day 1 and 80 mg/day on days 2–3) were analyzed. The regimens comprised 12 mg DEX on Day 1 and 8 mg/day on Days 2–4, administered intravenously; PALO or GRA administered intravenously on Day 1 together with DEX for less than 15 min, at least 30 min prior to cisplatin administration; and the dose of APR administered at least 60 min prior to cisplatin administration on Day 1, and the doses of APR administered before breakfast on Days 2 and 3.

### Cost

Cost analysis was performed from the healthcare payer perspective, and direct medical costs associated with CINV prevention and the additional medical fees incurred by CINV were estimated (Table 2). In Japan, patients are covered by NHI system, and the copayment of a patient is 10–30% of the total medical cost according to his/her age. The Ministry of Health, Labour, and Welfare determines prescribing drug prices and expenses for medical treatment and care and registers them in the NHI standard list to which national insurance is applicable. All costs in this study were assigned from the NHI Drug Price Standard listed in 2018 [15] and the Reimbursement Schedule of Social Insurance [16]. For rescue treatment, the costs associated with follow-up visits, the cost of test fees (biochemical tests [over 10 items], C-reactive protein, venous blood sampling), the cost of medications (prescription, preparation, and base dispensing fee if prepared at a hospital), and the cost of the intravenous drip infusion were included in the calculation. The cost associated with the administration of chemotherapy other than the antiemetic therapy would be the same in both groups, and this cost was not included in the calculation.

**Table 2:** Study parameter and ranges in one-way sensitivity analysis

Pramater	Values	Range	Ref
CINV related health state probabilities			
Complete response in acute phase in PALO regimen	0.918	95% CI	[7]
Complete response in delayed phase in PALO regimen	0.672	95% CI	[7]
Complete response in acute phase in GRA regimen	0.918	95% CI	[7]
Complete response in delayed phase in GRA regimen	0.591	95% CI	[7]
Costs	JPY (USD)	JPY (USD)	
Palonosetron 0.75 mg (IV)	14,972 (133.5)	7,486-14,972 (66.7-133.5)	[15]
Granisetoron 1 mg (IV)	1,273 (11.3)	590 -1,273 (5.26-11.3)	[15]
Aprepitantcapusule set (125 mg /80 mg /80 mg) (PO)	11,638.2 (103.8)		[15]
Dexametazone 2 mg (IV)	99 (0.883)		[15]
Dexametazone 4 mg (PO)	34 (0.303)		[15]
Blood test^b^	1,580 (14.1)		[16]
Internal medicine for rescue medication for PALO regimen	432 (3.85)	± 30%	[15, 18, 19]
Internal medicine for rescue medication for GRA regimen	5,956.5 (53.1)	± 30%	[15, 18, 19]
Infusion therapy for rescue medication	1,374 (12.3)	± 30%	[15, 16, 18, 19]
Total cost per acute CINV in PALO regimen	432 (3.85)		
Total cost per delayed CINV in PALO regimen	2,396 (21.36)		
Total cost per acute CINV in GRA regimen	5,956.5 (53.10)		
Total cost per delayed CINV in GRA regimen	10,221 (91.12)		
Utility values			
Complete protection	0.9	± 30%	[13, 14, 20–22]
Complete response at best	0.7	± 30%	[13, 14, 20–22]
Incomplete response	0.2	± 30%	[13, 14, 20–22]

*CINV* Chemotherapy induced nausea and vomitingn, *PALO* Palonosetron, *GRA* Granisetron, *CI* Confidence interval, *JPY* Japanese yen, *IV* Intravenous, *PO* Oral. Exchange rate, 1 USD = 112.17 JPY

IR in the acute or the delayed phase was taken to be the administration of additional antiemetic medication and fluid replacement as rescue medication. The drug cost of additional rescue treatment for CINV for each health state in the TRIPLE study was reported by Shimizu et al. [11]. In the trial, 5-HT_3_RAs were not administered additionally. In clinical practice, 5-HT_3_RAs are widely used for rescue treatment [17]. In this analysis, rescue treatment was set according to clinical practice and other previous studies instead of the cost result of the trial. The JSCO Clinical Practice Guidelines for Antiemesis in Oncology state that dopamine receptor antagonists, steroids, or 5-HT_3_RAs are useful as additional agents for breakthrough nausea or vomiting. Further, for additional medication, the guidelines recommend changing the 5-HT_3_RA to a different one from the 5-HT_3_RA used for prophylactic administration. Accordingly, ramosetron, a tablet that is highly useful in clinical settings because it breaks down in the mouth and has a mid-range price, was selected as the additional 5-HT_3_RA. In the acute phase, additional medication was administered orally. In the delayed phase, the patient was re-assessed at a medical institution, with examination, prescription, and infusion performed by a doctor and prescriptions made up in the hospital by a pharmacist. The three drugs used in rescue therapy, which were prescribed with reference to the cost-effectiveness studies of DEX and PALO in prophylactic antiemetic therapy by Oshima et al. [18] and Yamanishi et al. [19], were 1 tablet of metoclopramide 3 times a day for 5 days, one 4-mg tablet of dexamethasone twice a day for 5 days, and one tablet a day of the 5-HT_3_RA ramosetron for 5 days. However, the 5-HT_3_RA was not used after PALO administration. In addition, a transfusion of 500 mL of BFLUID® once a day for 2 days was given as intravenous feeding. Since there were no clinically relevant differences in the overall incidence of adverse events [7], in the present analysis, the costs relating to adverse reactions to the antiemetic treatment were not included in the totals. At the time of analysis, costs were calculated using the 2018 drug prices and medical fees and converted into dollars using the most recent annual exchange rate published by the OECD (1 USD = 112.17 JPY) [23].

### Utility

The utility value of the health state of CINV in Japanese has not been reported in clinical trials and there is no publicly available information. Sun et al. report a QoL evaluation using a visual analog scale according to the presence or absence of CINV by chemotherapy [20]. Including this report, according to previous CINV reports [13, 14, 21, 22], utility values were applied to the three health states. The following utility values were defined: 0.9 for CP with no significant nausea or vomiting; 0.2 for IR with nausea and vomiting; and 0.7 for CRB without vomiting or use of rescue medication. The health state in the acute period was set as 1 day (24 h), and the health condition of the delay period was set as 4 days (96 h). The sum of the 5-day Quality-adjusted life years (QALYs) was calculated using the following formula: QALYs = ([utility value (acute phase) × 1 d] + [utility value (delayed phase) × 4 d])/365 d. Since information available for estimating the utility value of CINV is insufficient, a sensitivity analysis was performed to examine the influence of these changes on the results.

### Cost-effectiveness analysis

This study was carried out in compliance with the Guideline for Economic Evaluation of Healthcare Technologies in Japan [24] and the Consolidated Health Economic Evaluation Reporting Standards Statement [25].

Cost-effectiveness was calculated from the costs incurred in antiemetic therapy and QALYs for 5 days following anticancer agent administration as a health outcome. The incremental cost-effectiveness ratio (ICER) of the base case was calculated. In this study, discounts were not applied during the 5-day short-term observation period. The threshold of willingness-to-pay (WTP) used 5,000,000 JPY (44,575 USD/QALY) defined by Shiroiwa et al. [26]. TreeAge Pro 2016 (TreeAge Software, Inc., Williamstown, MA, USA) was used for the analysis.

### Sensitivity analysis

For this study, a clinical decision analysis simulation model was constructed, and a hypothetical situation was defined with variables such as transition probabilities, utilities, and costs. One-way and probabilistic sensitivity analyses were carried out to assess the uncertainty and robustness of this model by evaluating the effects of differing model parameters. In the one-way sensitivity analysis, the efficacy ratio of antiemetic therapy was examined in the range of the 95% confidence interval of the effectiveness in the TRIPLE study. Changes were examined with the utility value and cost in the range ± 30%, although the cost of GRA was examined up to the cheapest price of the generic drug, and the cost of PALO is unlikely to increase with future drug price revision, and only the lower 50% limit was considered because only the possibility of a price decrease was considered. A probabilistic sensitivity analysis (PSA) was carried out to examine changes in data that included uncertainty of the base case and to investigate the robustness of the results, and the effects on the ICER were examined. In PSA, a 10,000-sample Monte Carlo simulation was performed with transition probability and utility value set as having beta distributions and cost as having a gamma distribution. The probability of the ICER being less than the WTP value was determined from the cost-effectiveness acceptability curve (CEAC). Additionally, a threshold analysis was performed to determine the cost-effectiveness price of PALO for WTP of 5,000,000 JPY.

## Results

### Base case results

Expected costs for drug expenses of antiemesis treatment and utility values were estimated. The costs of antiemetic therapy per course were calculated to be 27,406 JPY (244.33 USD) with the PALO regimen and 13,707 JPY (122.20 USD) with the GRA regimen. The medical expenses were 1,580 JPY (14.09 USD) for blood testing, 590 JPY (5.25 USD) for pharmacy costs, and 1,374 JPY (12.25 USD) for supplementary nutrition infusion a single time with both regimens. The costs of oral agents for rescue medication were calculated to be 432 JPY (3.85 USD) with the PALO regimen and 5,957 JPY (53.1 USD) with the GRA regimen. The base case results over 5 days after cycle 1 of chemotherapy are shown in Table 3. For the PALO regimen, the incremental effect per person was 0.0006452 QALY, the incremental cost was 10,455 USD (93.21 USD), and the ICER was 16,204,591 JPY QALY (144,465 USD/QALY). The economic results with the two strategies for each cost category are shown in Table 4. Although the cost for rescue medication in the PALO regimen was low, the total cost was higher than in the GRA regimen.

**Table 3:** Base-case results

Strategy	Cost JPY (USD)	Incrmental Cost JPY (USD)	QALY	Incremental QALY	ICER JPY/QALY (USD/QALY)
PALO regimen	30,348 (270.55)	10,455 (93.21)	0.009598	0.000645208	16,204,591 (144,465)
GRA regimen	19,893 (177.35)		0.008952		

*JPY* Japanese yen, *QALY* Quality-adjusted life year, *ICER* Incremental cost-effectiveness ratio. Exchange rate, 1 USD = 112.17 JPY

**Table 4:** Economic results of the two strategies per each cost category

Strategy	Cost category JPY (USD)			
	Total cost	Rescue medication	Medical fee	Prophyraxis
PALO Regimen	30,348 (270.55)	617 (5.50)	949 (8.46)	27,406 (244.33)
GRA Regimen	19,893 (177.35)	3,290 (29.33)	1,183 (10.55)	13,707 (122.20)

*JPY* Japanese yen. Exchange rate, 1 USD = 112.17 JPY

### Sensitivity analysis

**Fig. 2:**
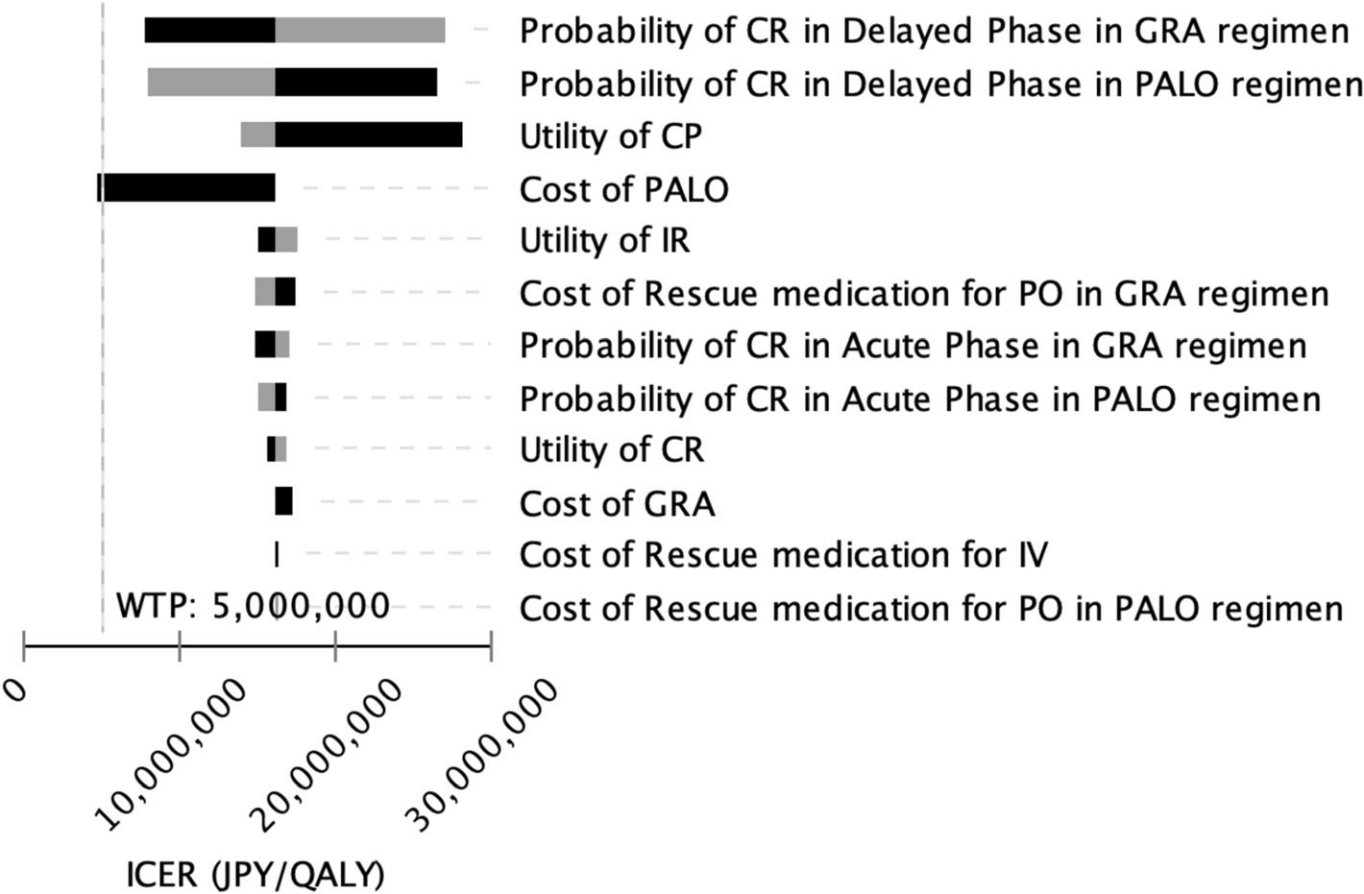
Tornado diagram for one-way sensitivity analyses. WTP: Willingness to pay; ICER: incremental cost-effectiveness ratio; JPY: Japanese yen; QALY: quality-adjusted life year; CR: complete response; PALO: palonosetron; GRA: granisetron; CP: complete protection; IR: incomplete response; PO: oral; IV: ntravenous

**Fig. 3:**
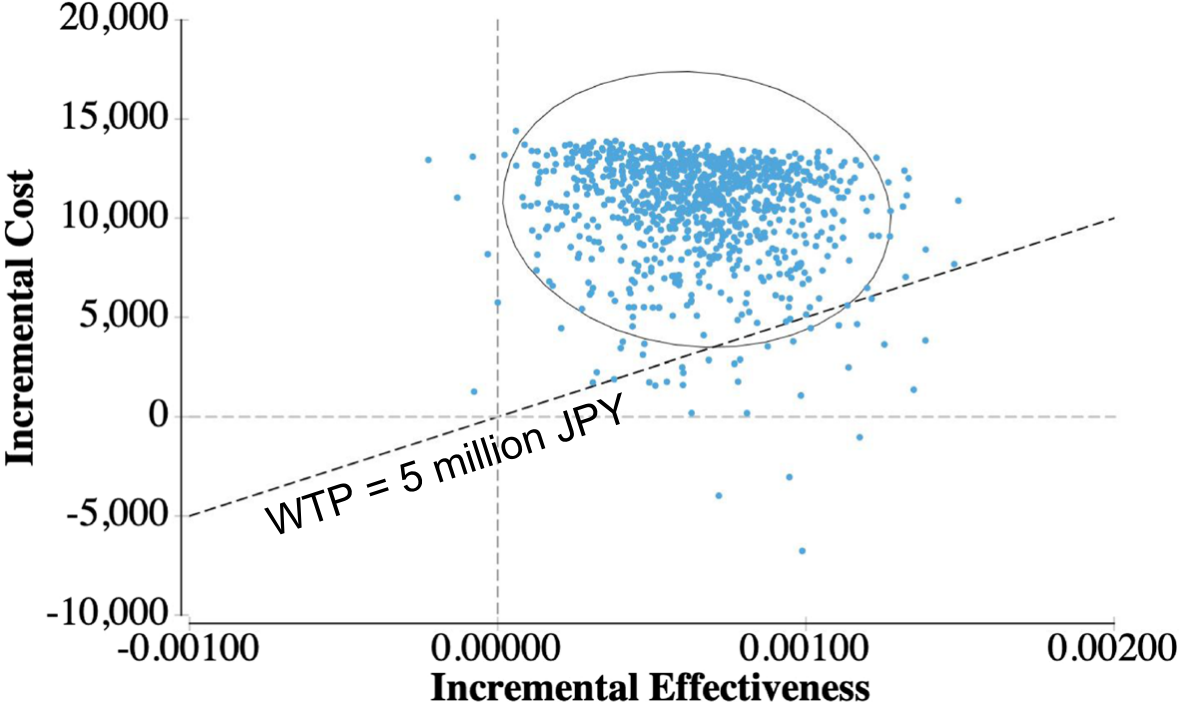
Incremental cost-effectiveness plane for probabilistic sensitivity analyses. WTP: Willingness-to-pay; JPY: Japanese yen

**Fig. 4:**
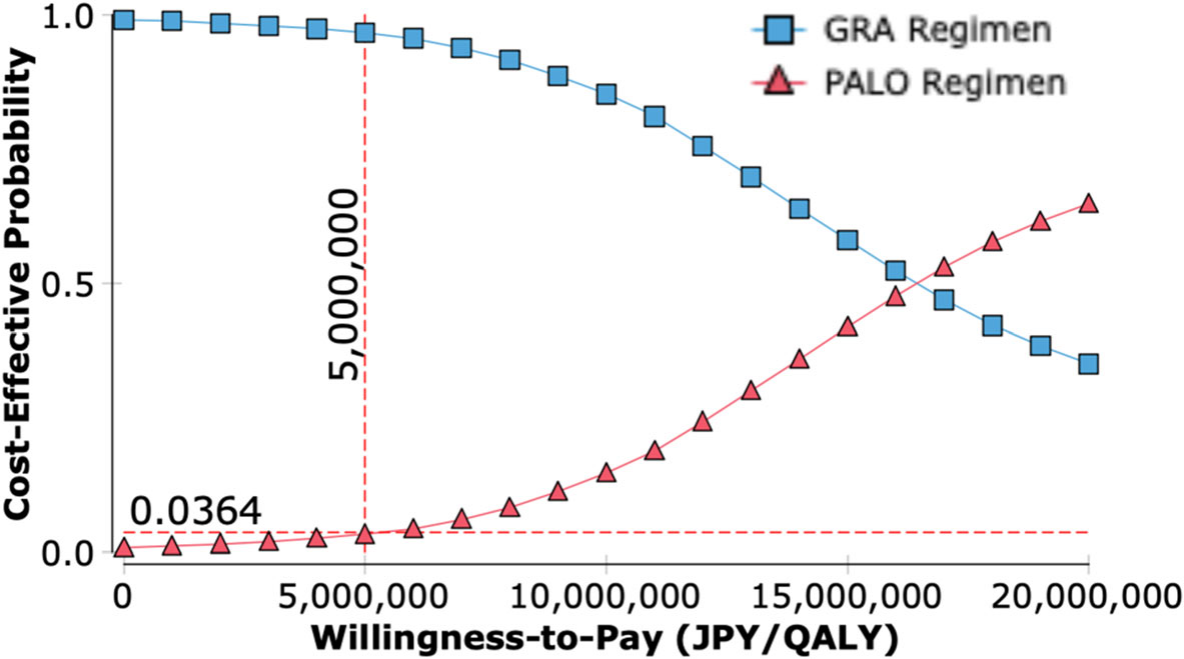
Cost-effectiveness acceptability curve for probabilistic sensitivity analyses. PALO: palonosetron; GRA: granisetron; JPY: Japanese yen

**Fig. 5:**
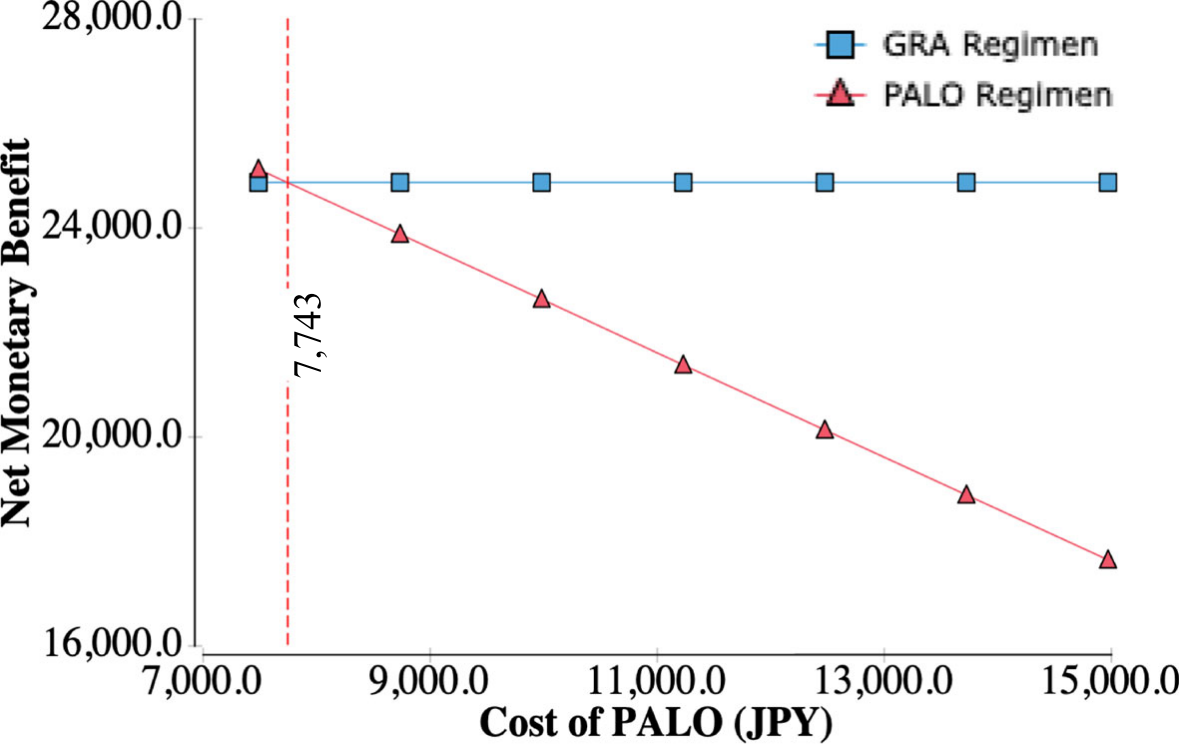
Results of the threshold analysis for the cost of palonosetron. PALO: palonosetron; GRA: granisetron; JPY: Japanese yen

The results of the one-way sensitivity analysis are shown in a tornado diagram (Fig. 2). This diagram shows the effects of uncertainly on the ICER for each parameter. The largest effect of PALO on the increase in the ICER was on the CR rate in the delayed phase in the PALO regimen and the CR rate in the delayed phase in the GRA regimen, and the greatest ICER was approximately 27,120,000 JPY/QALY (242,000 USD/QALY) and 26,460,000 JPY/QALY (236,000 USD/QALY). With a reduction of 50% in the drug price for PALO, the ICER decreased to approximately 4,600,000 JPY/QALY (41,000 USD/QALY). Maximums in the ICER due to other parameters were less than 20,000,000 JPY/QALY (178,300 USD). An incremental cost-effectiveness plane and a CEAC were drawn from the results of the PSA. Many points in this analysis existed in the northeastern quadrant (i.e., more effective and more expensive) and existed above the diagonal line showing ICER of 5,000,000 JPY per QALY (Fig. 3). At a WTP threshold of 5,000,000 JPY, the PALO regimen had an acceptability of 3.64% (Fig. 4). The results of the threshold analysis are shown in Fig. 5. The estimated threshold value of PALO was 7743 JPY (69.03 USD) when PALO in a triplet antiemetic regimen was compared with GRA in the base case.

## Discussion

The present study used the results of the TRIPLE study to perform a cost-utility analysis within the Japanese health care system from the health care payer perspective. This is the first report in which the ICER was calculated with QALYs of the antiemetic triplet regimen of PALO in combination with APR and DEX compared to that of GRA with APR and DEX for cisplatin-containing HEC. The results showed the incremental effect per person to be 0.0006452 QALYs, and the incremental cost was 10,455 JPY (93.21 USD). The base case ICER was approximately 16,200,000 JPY QALY (144,000 USD/QALY). No consensus exists regarding the threshold for acceptable cost per QALY ratios in Japan’s health policy. Therefore, the common WTP threshold of 5,000,000 JPY/QALY from a previous study was adopted. Based on this standard, it was concluded that prophylaxis with PALO was not cost-effective for HEC in Japan. This conclusion was considered robust based on the results of the sensitivity analyses. In the one-way sensitivity analyses, the clinical trial results were examined on the basis of the 95% confidence interval. The results indicate that the CR rate in the delayed phase had the greatest effect on the ICER. The onset of delayed-phase CINV was thus attributable to the patients’ decreased QoL and increases in costs associated with rescue therapy, such as medical consultation fees, test fees, and drug costs. In addition, a 50% reduction in the drug price of PALO reduced the ICER to approximately 46,000,000 JPY/QALY (41,000 USD/QALY), bringing it to below the WTP value. Using the results of the threshold analysis, the acceptable price of PALO was estimated to be 7743 JPY (69.03 USD). This price is 51.7% of the current price of 14,972 JPY. Therefore, a price reduction is necessary for PALO to be considered cost-effective by commonly applied thresholds. The cost of drugs, with the arrival of inexpensive generic drugs, will make a major contribution to the cost-effectiveness of the therapy. In the PSA, with the WTP at 5,000,000 JPY, the probability of the PALO regimen being judged to be cost-effective was only 3.64%. Even taking the diversity of diagnostic patterns and the individual differences of patients into account, antiemetic therapy using PALO was shown to be less cost-effective. The JSCO Clinical Practice Guidelines for Antiemesis in Oncology recommend the use of PALO with HEC containing 50 mg/m^2^ or more cisplatin from the point of view of efficacy. However, similar to the report of Shimizu et al., the results of the present study indicate that there is still concern about the cost-effectiveness of antiemetic therapy using PALO at this time.

The results of the present study can be compared to the results of prior studies in other countries. Avritscher et al. [9] compared the cost-effectiveness in the US medical care system of the triplet regimen of PALO, APR, and DEX with a control of combined ondansetron and DEX in breast cancer patients who had undergone anthracycline and cyclophosphamide treatment using a Markov model. The results showed that the ICER for the triplet regimen including PALO was 115,490 USD (approx. 13,000,000 JPY). Du et al. [10] compared the cost-effectiveness in the Chinese medical care system of a doublet regimen of PALO and DEX against a control of ondansetron or GRA and DEX using a decision tree, and they reported the ICER of PALO to be 167,914 USD (approx. 19,000,000 JPY). Cawston et al. reported the cost-effectiveness of a combination drug (NEPA: netupitant plus palonosetron), comparing the combination with APR and PALO using a Markov model in the UK medical care system. They reported that NEPA was cost-effective for preventing CINV associated with highly or moderately emetogenic chemotherapy in the UK [27]. Restelli et al. reported the cost-effectiveness of NEPA, comparing the combination with NK1-RA (APR or fosaprepitant) and 5-HT3RA (PALO or ondansetron) in patients receiving highly or moderately emetogenic chemotherapy using a Markov model in the Italian medical care system. The results showed that NEPA was more effective and less expensive [28]. The present study compared a triplet regimen of APR, DEX, and PALO against a control of APR, DEX, and GRA in the Japanese medical care system. The data source was Japanese patients undergoing cisplatin-containing HEC from the TRIPLE study. Thus, the present study examined different types of cancer and chemotherapy from Avritscher et al. [9] and differs from Du et al. [10] in the doublet regimen of APR, and it is the first to report the cost-utility that evaluated QoL of PALO in comparison to GRA in triplet regimens containing APR for cisplatin-containing HEC. The fact that calculations were made using the unit price from each country, the differences between countries in drug prices and insurance systems, and the differences in the analysis models are reflected in the results of these studies, but all of them indicate that the cost-effectiveness of antiemetic therapy using PALO is not favorable. The drug price for PALO in Japan is over five times that of GRA, and it may be assumed that the same results were obtained, since there is the same or greater price difference in the USA and China. With the dramatic rises in drug prices in the past several decades, the use of new treatments should be tailored to those patients who are likely to benefit. For expensive PALO used for HEC as well, it is necessary to optimize prophylactic antiemetic therapy according to individual patient risk.

The limitations of the present study are as follows. First, the utility values in the analysis model conformed to prior studies, but they were based on data measured in other countries. It is highly likely that differences will be seen between the health care systems of different countries and regions, but since it is very difficult to evaluate the difference in utility values between Japan and other countries, the utility value patterns were assumed to be the same in Japan and other countries. The results of the one-way sensitivity analysis indicated that changes in the utility values did not greatly impact the results. The second limitation is that the present study analyzed outpatient chemotherapy on the basis of the TRIPLE study, but many hospitals that carry out inpatient chemotherapy have adopted a diagnosis procedure combination (DPC) payment system. Since DPC does not reflect differences in the costs of antiemetic therapy, calculation on the basis of fee for service should be performed. Shimizu et al. investigated the rescue treatment cost and total hospitalization cost of the TRIPLE study, but the results, such as shorter hospital stay and lower readmission rate due to CINV suppression, were not evaluated. These results are needed to evaluate differences in antiemetic outcomes in DPC systems. Because of the lack of data and evidence, the analysis was not able to examine inpatients. However, there is no evidence for these in Japan and studies with these results are awaited. Once the results are available, the model can incorporate the cost of hospitalization for DPC and analyze inpatient chemotherapy as well as outpatient chemotherapy. The third limitation is that the present study used only data from first-time chemotherapy.

This study showed the ICER of a triplet regimen of PALO, APR, and DEX for prevention of CINV in patients receiving cisplatin-containing HEC on the basis of the Japanese medical insurance system. In the future, it will be necessary to search for other cost-effective antiemetics such as olanzapine, and the result of this study is useful. Optimizing pricing of expensive medicines based on cost-effectiveness is an important financial issue in Japan. Based on the present findings, the Japanese government, as for other clinical interventions, may need to adjust the price of PALO in antiemetic therapy.

## Conclusion

The use of PALO instead of GRA for prevention of CINV in patients receiving HEC through the Japanese health insurance system is not cost-effective at this time. The cost of drugs, with the arrival of inexpensive generic drugs, will make a major contribution to its cost-effectiveness.

## Data Availability

The datasets used and/or analyzed during the current study are available from the corresponding author on reasonable request.

## Abbreviations

5-HT3RA: 5-hydrotryptamine-3 receptor antagonist
APR: Aprepitant
CEAC: Cost-effectiveness acceptability curve
CINV: Chemotherapy-induced nausea and vomiting
CP: Complete protection
CR: Complete response
CRB: Complete response at best
DEX: Dexamethasone
DPC: Diagnosis procedure combination
GRA: Granisetron
HEC: Highly emetogenic chemotherapy
ICER: Incremental cost-effectiveness ratio
IR: Incomplete response
JPY: Japanese yen
JSCO: Japan Society of Clinical Oncology
NHI: National health insurance
PALO: Palonosetron
PSA: Probabilistic sensitivity analysis
QALYs: Quality-adjusted life years
QoL: Quality of life
USD: US dollar
WTP: Willingness -to-pay
